# A new genus and species of foliicolous lichen in a new family of *Strigulales* (*Ascomycota*: *Dothideomycetes*) reveals remarkable class-level homoplasy

**DOI:** 10.1186/s43008-019-0026-2

**Published:** 2020-02-05

**Authors:** Shu Hua Jiang, David L. Hawksworth, Robert Lücking, Jiang Chun Wei

**Affiliations:** 1 State Key Laboratory of Mycology, Institute of Microbiology, Chinese Academy of Sciences, Beijing, China; 2 Department of Life Sciences, The Natural History Museum, Cromwell Road, London, SW7 5BD UK; 3 Comparative Plant and Fungal Biology, Royal Botanic Gardens, Kew, Surrey, TW9 3DS UK; 4 Jilin Agricultural University, Changchun, 130118 Jilin Province China; 5 Botanischer Garten und Botanisches Museum, Freie Universität Berlin, Königin-Luise-Straße 6–8, 14195 Berlin, Germany; 6 University of Chinese Academy of Sciences, Beijing, China

**Keywords:** *Phylloblastia*, *Phyllocratera*, *Phylloporis*

## Abstract

Phylogenetic analysis of some foliicolous lichens collected in Hainan Province, China, revealed a new lineage morphologically similar to *Porina* but phylogenetically related to *Strigulaceae* (*Dothideomycetes*), differing from the latter in ascus type. The monospecific genus *Tenuitholiascus* gen. nov. is introduced for the single species, *T. porinoides* sp. nov., which is placed in the new, monogeneric family *Tenuitholiascaceae*, sister to *Strigulaceae* in *Strigulales*. The new taxon closely resembles the genus *Porina* in external morphology and ascospore type, as well as the thin-walled asci and unbranched paraphyses. Yet, it is entirely unrelated to the latter, which belongs in class *Lecanoromycetes* in the order *Gyalectales*.

## INTRODUCTION

Foliicolous lichens are widespread in the tropics and extraordinarily diverse (Santesson 1952; Lücking 2001, 2008). Their small size and their tendency to occur mixed with other foliicolous lichens and non-lichenized fungi in minute communities, and in some cases their growth under the host plant cuticle, has rendered a re-appraisal of the relationships of these fungi by molecular phylogenetic methods technically difficult. Yet, some chiefly foliicolous lineages such as *Gomphillaceae*, *Pilocarpaceae*, *Porinaceae*, and the genus *Chroodiscus* (*Graphidaceae*) have been partly studied phylogenetically (Lücking et al. 2004; Andersen and Ekman 2005; Baloch and Grube 2006, 2009; Papong et al. 2009).

The order *Strigulales* in class *Dothideomycetes* was established by Lücking et al. (in Hyde et al. 2013) for the single family *Strigulaceae*, in an updated phylogeny which showed that the family was separate from other clades recognized as orders. Four genera (*Flavobathelium*, *Phyllobathelium*, *Phyllocratera*, *Strigula*) were accepted in the family at that time, but with the inclusion of the poorly known, monospecific genus *Oletheriostrigula* (Huhndorf and Harris 1996), that number has grown to five (Jaklitsch et al. 2016; Lücking et al. 2017). Members of this lineage are mostly found on leaves, with the most speciose genus being *Strigula*, and more rarely on bark and rocks, mostly in tropical to subtropical habitats, with very few species extending into temperate regions (Harris 1995; Roux et al. 2004; Aptroot and Moon 2014). *Strigulales* have been analysed molecularly in various studies (Nelsen et al. 2009, 2011a), particularly focusing on species delimitation in foliicolous representatives of *Strigula* in tropical Asia (Jayalal et al. 2013; Krishnamurthy and Subramanya 2016; Jiang et al. 2016, 2017a, 2017b; Krishnamurthy and Kumar 2017).

As part of a phylogenetic revision of species of *Strigula* s.lat. and similar taxa, we came across a novel lineage resembling species of *Porina* but clustering with *Strigulales* in *Dothideomycetes*. The diagnostic feature of this new lineage is the thin-walled ascus apex, an unusual feature for *Dothideomycetes*. Given that *Strigulales* and the related families *Acrospermaceae*, *Dyfrolomycetaceae*, and *Kirschsteiniotheliaceae* all have bitunicate asci with a thin but distinct tholus and ocular chamber (Huhndorf and Harris 1996; Lücking 2008; Hyde et al. 2013), we recognize this new taxon as a new genus (*Tenuitholiascus*) in a new family (*Tenuitholiascaceae*). Initially we also considered introducing a new order, but since families with different ascus types have been shown to be closely related and included in single orders in other instances, such as *Lecanorales* in *Lecanoromycetes* (Miadlikowska et al. 2014), we include the new family in the order *Strigulales*, related to the orders *Acrospermales* (Minter et al. 2007), *Dyfrolomycetales* (Hyde et al. 2013), and *Kirschsteiniotheliales* (Hernández-Restrepo et al. 2017).

## MATERIAL AND METHODS

### Material examined

The interesting specimens regarding the new family were all collected from Hainan province in China. All are preserved in the Fungarium-Lichenum of the Institute of Microbiology, Chinese Academy of Sciences (HMAS-L).

## METHODS

### Phenotypic analyses

A LEICA M125 dissecting microscope (Leica Microsystems, Singapore) was used for the morphological studies, and a Zeiss Axioscope2 (Carl Zeiss, Göttingen) compound microscope for the anatomical studies and measuring the size. Photographs were obtained with an AxioCam MRc5 connected to a Zeiss Imager A2-M2 microscope (Carl Zeiss, Göttingen) for microscopic features. Thin-layer chromatography (TLC) (Orange et al. 2001) was employed for the detection of lichen substances. The features of the ascus apex were revealed using Lugol’s solution without pre-treatment by KOH (Baral 1987).

### Genotypic analyses

#### DNA extraction and PCR amplification

All newly collected fresh specimens were subjected to DNA extraction (Table 1), for which a modified CTAB method (Rogers and Bendich 1988) was used. DNA, suspended in ddH_2_O, was amplified by the polymerase chain reaction (PCR). Partial nuclear ribosomal small subunit SSU sequences were amplified and sequenced using combinations of the primers SF5-CAATTGGAGGGCAAGTCTGG and SR5-CCAAGAGATCCGTTGTTGAAAG (in this study). A portion of the fungal nuclear ribosomal large subunit LSU was amplified and sequenced using primers ITS3 (White et al. 1990) and LR72-TACTACCACCAAGATCTGCAC. Partial TEF1-α sequences were generated using the primers TEF1a-983 F (Rehner and Buckley 2005) and TEF1a-1567R-HTL (Nelsen et al. 2011a). The second largest subunit of RNA polymerase II, RPB2, was amplified and sequenced using primers: fRPB2-5F-GAYGAYMGWGATCAYTTYGG and fRPB2-7cR- CCCATRGCTTGYTTRCCCAT (Liu et al. 1999).

**Table 1:** Sequences for molecular phylogenetic analysis in *Strigulales*. The new sequences generated were in bold

Species	Species No.	GenBank Accession No. (LSU, SSU, TEF1-α, RPB2)			
		LSU	SSU	*TEF1-α*	*RPB2*
*Flavobathelium epiphyllum*	MPN67	GU327717	JN887382	JN887423	–
*Phyllobathelium anomalum*	MPN242	GU327722	JN887386	JN887430	–
***Tenuitholiascus porinoides*** **sp. nov.**	**HMAS_L0139638**	**MK206259**	**MK352441**	**MK273106**	**MK273134**
***T. porinoides*** **sp. nov.**	**HMAS_L0139639**	**MK206258**	**MK352442**	**MK273105**	**MK273133**
***T. porinoides*** **sp. nov.**	**HMAS_L0139640**	**MK206260**	**MK352443**	**MK273107**	**MK273135**
***Strigula acuticonidiarum***	**HMAS_L0138045**	**MK206236**	**MK206217**	**MK273083**	**MK273111**
***S. guangxiensis***	**HMAS_L0138040**	**MK206256**	–	**MK273103**	**MK273131**
***S. guangxiensis***	**HMAS_L0138041**	**MK206257**	–	**MK273104**	**MK273132**
***S.*** **cf.** ***macaronesica***	**HMAS_L0130615**	**MK206251**	**MK206230**	**MK273098**	**MK273126**
***S.*** **cf.** ***macaronesica***	**HMAS_L0139260**	**MK206252**	**MK206231**	**MK273099**	**MK273127**
***S. macrocarpa***	**HMAS_L0141394**	**MK206240**	**MK206221**	**MK273087**	**MK273115**
***S. macrocarpa***	**HMAS_L0139289**	**MK206241**	**MK206222**	**MK273088**	**MK273116**
*S. nemathora*	MPN72	JN887405	JN887389	JN887433	–
***S. nitidula***	**HMAS_L0139358**	**MN788374**	**MN788375**	**MN793983**	**MN793982**
***S. sinoaustralis***	**HMAS_L0137204**	**MK206249**	–	**MK273096**	**MK273124**
***S.*** **cf.** ***smaragdula***	**HMAS_L0141395**	**MK206234**	**MK206215**	**MK273081**	**MK273109**
***S.*** **cf.** ***smaragdula***	**HMAS_L0141396**	**MK206233**	**MK206214**	**MK273080**	**MK273108**
***S.*** **cf.** ***smaragdula***	**HMAS_L0139166**	**MK206235**	**MK206216**	**MK273082**	**MK273110**
***S. univelbiserialis***	**HMAS_L0137657**	**MK206243**	**MK206224**	**MK273090**	**MK273118**
***S. univelbiserialis***	**HMAS_L0137658**	**MK206245**	**MK206226**	**MK273092**	**MK273120**
***S. univelbiserialis***	**HMAS_L0137659**	**MK206242**	**MK206223**	**MK273089**	**MK273117**
***S. univelbiserialis***	**HMAS_L0137660**	**MK206244**	**MK206225**	**MK273091**	**MK273119**

Photosymbionts of selected specimens were also analyzed phenotypically and molecularly besides phenotype (Table 2). ITS nrDNA sequences of the algal partners were amplified and sequenced using the primers nr-SSU-1780-59 and nr-LSU-0012-39 (Piercey-Normore and Depriest 2001).

**Table 2:** Sequences of photobiont *Trentepohliaceae*. The new sequences generated were in bold

Species	Species No.	GenBank Accession No.(ITS)
*Cephaleuros expansus*	GD1318	KX586811
*Cephaleuros karstenii*	DZ1309	KX586781
*Cephaleuros karstenii*	DZ1312	KX586784
***Cephaleuros*** **sp. from** ***Strigula*** **sp.**	**HMAS_L0130622**	**MK211171**
***Cephaleuros*** **sp. from** ***S.*** cf. ***smaragdula***	**HMAS_L0141395**	**MK211172**
*Phycopeltis aurea*	YN1220	KP067280
***Phycopeltis*** **sp*.*** **from** ***Tenuitholiascus porinoides*** **sp. nov.**	**HMAS_L0139638**	**MK211174**
***Phycopeltis*** **sp*.*** **from** ***T. porinoides*** **sp. nov.**	**HMAS_L0141346**	**MK211173**
*Phycopeltis* sp*.*	YN1202	KP067279
*Trentepohlia* sp*.*	DS22	KC489115
*Trentepohlia* sp*.*	SAG 118.80	KM020078
*Trentepohlia* sp*.*	TreFl54	KC489121
*Ulva tepida*	PR18	KT374011

PCR reactions were carried out in 25 μl reaction volumes and the components used were: 2 μl total DNA, 1 μl each primer (10 μM), 12.5 μl 2 × Taq MasterMix, 8.5 μl ddH_2_O. Amplification was performed using a Biometra T-Gradient thermal cycler. Cycling parameters for LSU, ITS and SSU were set to an initial denaturation at 95 °C for 5 min, followed by 35 cycles of denaturation at 94 °C for 30 s, annealing at 54 °C for 30 s, extension at 72 °C for 1 min, and a final extension at 72 °C for 10 min. PCR amplifications of TEF1-α were initiated with a 2 min denaturation at 94 °C. The annealing temperature in the first amplification cycle was 66 °C, which was subsequently incrementally reduced by 1 °C per cycle over the next 9 cycles. An additional 30 amplification cycles were then performed, each consisting of 30 s denaturation at 94 °C, a 30 s annealing step at 56 °C, and a 1 min extension at 72 °C, concluding with a 10 min incubation at 72 °C (Rehner and Buckley 2005). The PCR conditions of RPB2 included: initial denaturation at 95 °C for 5 min; 35 cycles of 1 min at 95 °C, 2 min at 50 °C, an increase of 1 °C/5 s to 72 °C, and 2 min at 72 °C; and a 10 min incubation at 72 °C (Liu et al. 1999). PCR products were checked on 0.8% agarose electrophoresis gels stained with ethidium bromide and then sent to the sequencing facilities of Majorbiology (Changping district, Beijing, China), for sequencing.

#### Sequence alignment and phylogenetic analyses

For the mycobionts, sequences generated from different primers (Table 1) were analyzed with others obtained from the GenBank (Table 3). To determine the exact placement of the new lineage, a three-locus (SSU, LSU, and TEF-α) dataset was compiled in which sequences were aligned with those retrieved from GenBank covering the main groups of the class *Dothideomycetes*. In total, 109 ingroup taxa were used together with four outgroup taxa representing *Arthoniomycetes*. Further, a four-locus (SSU, LSU, TEF-α, and RPB2) dataset was also analysed with those retrieved from GenBank covering *Dothideomycetes* and *Lecanoromycetes* of the phylum *Ascomycota*, with three outgroup taxa representing *Basidiomycota* (Table 3). For the datasets, we only used specimens with the highest number of available markers. Each partition of LSU, SSU, TEF1-α, and RPB2 was aligned independently and then the alignments were concatenated for multi-locus analyses. Generated ITS sequences of algal partners were aligned with eight samples of *Trentepohliaceae* from GenBank (Table 2). All sequences were aligned with MAFFT v7.402 (Katoh and Toh 2010).

**Table 3:** Other sequences retrieved from GenBank for phylogenetic analysis

Species	GenBank Accession No. (LSU, SSU, *TEF1-α,* RPB2)			
	LSU	SSU	*TEF1-α*	RPB2
*Acarospora cervina*	AY640941	AY640982	–	AY641021
*Acarospora laqueata*	AY640943	AY640984	–	AY641024
*Acrospermum adeanum*	EU940104	EU940031	–	EU940320
*Acro. compressum*	EU940084	EU940012	–	EU940301
*Acro. gramineum*	EU940085	EU940013	–	EU940302
*Aigialus grandis*	GU301793	GU296131	–	GU371762
*Aigialus parvus*	GU301795	GU296133	GU349064	GU371771
*Aliquandostipite khaoyaiensis*	GU301796	AF201453	GU349048	FJ238360
*Anisomeridium ubianum*	GU327709	JN887379	–	–
*Apiosporina collinsii*	GU301798	GU296135	GU349057	–
*Armillaria mellea*	AY700194	AY787217	AY881023	AY780938
*Aquasubmersa japonica*	LC061588	LC061583	LC194385	LC194422
*Arthopyrenia salicis*	AY538339	AY538333	–	–
*Ascocratera manglicola*	GU301799	GU296136	–	GU371763
*Asterina cestricola*	GU586215	GU586209	–	–
*As. fuchsiae*	GU586216	GU586210	–	–
*As. phenacis*	GU586217	GU586211	–	–
*As. weinmanniae*	GU586218	GU586212	–	–
*As. zanthoxyli*	GU586219	GU586213	–	–
*Aureobasidium pullulans*	DQ470956	DQ471004	DQ471075	DQ470906
*Botryobambusa fusicoccum*	JX646809	JX646826	–	–
*Botryosphaeria agaves*	JX646808	JX646825	–	–
*Botryosphaeria dothidea*	DQ678051	DQ677998	DQ767637	DQ677944
*Calocera cornea*	AY701526	AY771610	AY881019	AY536286
*Cladonia caroliniana*	AY584640	AY584664	DQ782888	AY584684
*Cladonia stipitata*	DQ973026	DQ973003	–	DQ973087
*Coccocarpia erythroxyli*	DQ883800	DQ883791	DQ883775	DQ883756
*Delitschia didyma*	DQ384090	AF242264	–	–
*Delitschia winteri*	DQ678077	DQ678026	DQ677922	DQ677975
*Dendrographa decolorans*	AY548815	AY548809	DQ883725	
*Dothidea hippophaeos*	DQ678048	U42475	DQ677887	DQ677942
*D. insculpta*	DQ247802	DQ247810	DQ471081	AF107800
*D. sambuci*	AY544681	AY544722	DQ497606	KT216559
*Dothiora cannabinae*	DQ470984	DQ479933	DQ471107	DQ470936
*Dyfrolomyces rhizophorae*	GU479799	GU479766	GU479860	–
*Dyfrolomyces tiomanensis*	KC692156	KC692155	KC692157	–
*Elsino centrolobi*	DQ678094	DQ678041	DQ677934	–
*E. phaseoli*	DQ678095	DQ678042	DQ677935	–
*E. veneta*	DQ767658	DQ767651	DQ767641	–
*Falciformispora lignatilis*	GU371826	GU371834	GU371819	–
*Fal. senegalensis*	KF015627	KF015634	KF015688	KF015716
*Fal. tompkinsii*	KF015625	KF015640	KF015685	KF015718
*Flavoparmelia caperata*	AY584639	AY584663	DQ883763	AY584685
*Fomitopsis pinicola*	AY684164	AY705967	AY885152	AY786056
*Gibbera conferta*	GU301814	GU296150	GU349041	–
*Gloniopsis praelonga*	FJ161195	FJ161154	FJ161103	FJ161113
*Glonium circumserpens*	FJ161200	FJ161160	FJ161108	FJ161126
*Glonium stellatum*	FJ161179	FJ161140	FJ161095	–
*Guignardia gaultheriae*	DQ678089	–	–	
*Heterodermia vulgaris*	KX512857	DQ883789	DQ883773	DQ883754
*Hysteropatella clavispora*	AY541493	DQ678006	DQ677901	DQ677955
*Jahnula aquatica*	EF175655	EF175633	–	–
*J. bipileata*	EF175657	EF175635	–	–
*Kirschsteiniothelia aethiops*	AY016361	AY016344	DQ677884	DQ470914
*Kirschsteiniothelia lignicola*	HQ441568	HQ441569	–	–
*Lecanactis abietina*	AY548812	AY548805	–	
*Lecanora contractula*	DQ986746	DQ986741	–	DQ992428
*Lepidosphaeria nicotiae*	DQ678067	–	DQ677910	DQ677963
*Lichenoconium aeruginosum*	HQ174269	–	–	–
*L. erodens*	HQ174267	–	–	–
*L. lecanorae*	HQ174263	–	–	–
*L. usneae*	HQ174265	–	–	–
*Lichenothelia calcarea*	KC015061	KC015081	–	–
*Lichenothelia convexa*	KC015068	KC015083	–	–
*Lindgomyces breviappendiculata*	AB521748	AB521733	–	–
*Lindgomyces ingoldianus*	AB521736	AB521719	–	–
*Lobariella pallida*	DQ883797	DQ883788	DQ883772	DQ883753
*Lophiotrema neoarundinaria*	AB524596	AB524455	AB539110	AB539097
*Macrophomina phaseolina*	DQ678088	DQ678037	DQ677929	KX463996
*Massariosphaeria grandispora*	GU301842	GU296172	GU349036	GU371725
*Massariosphaeria typhicola*	GU301844	GU296174	–	GU371795
*Megalotremis verrucosa*	GU327718	JN887383	–	–
*Microthyrium microscopicum*	GU301846	GU296175	GU349042	GU371734
*Microxyphium aciculiforme*	GU301847	GU296176	GU349045	GU371736
*Microxyphium theae*	GU301849	GU296178	GU349060	–
*Myelochroa aurulenta*	EF042917	DQ973001	–	DQ973070
*Myriangium duriaei*	DQ678059	AY016347	DQ677900	DQ677954
*Myriangium hispanicum*	GU301854	GU296180	GU349055	GU371744
*Mytilinidion resinicola*	FJ161185	FJ161145	–	–
*Mytilinidion scolecosporum*	FJ161186	FJ161146	FJ161102	FJ161121
*Natipusilla bellaspora*	JX474863	JX474868	–	–
*N. decorospora*	HM196369	HM196376	–	–
*N. limonensis*	HM196370	HM196377	–	–
*N. naponensis*	HM196372	HM196379	–	–
*Neofusicoccum parvum*	AY928045	EU673151	–	FJ900618
*Neofusicoccum ribis*	DQ678053	DQ678000	DQ677893	EU339554
*Oedohysterium insidens*	GQ221882	GU323190	–	GU371785
*Ophiosphaerella sasicola*	AB524599	AB524458	AB539111	AB539098
*Parmotrema austrosinense*	DQ912338	DQ912315	–	DQ912386
*Peltigera degenii*	KX869856	AY584681	DQ782897	–
*Phaeotrichum benjaminii*	AY004340	AY016348	DQ677892	DQ677946
*Phyllosticta citricarpa*	GU301815	GU296151	GU349053	
*Physconia muscigena*	DQ912344	DQ912321	–	DQ912393
*Platismatia glauca*	KJ766626	KJ766768	–	DQ912388
*Pleopsidium chlorophanum*	DQ842017	DQ525541	DQ782920	DQ525442
*Pleopsidium gobiense*	DQ883698	DQ525573	DQ883804	DQ525452
*Protoparmeliopsis muralis*	KJ766634	–	–	KU935052
*Pseudotetraploa curviappendiculata*	AB524608	AB524467	–	–
*Rasutoria tsugae*	EF114705	EF114730	–	–
*Roccella fuciformis*	AY584654	AY584678	–	
*Roccella montagnei*	GU138014	AF110341	–	
*Roussoella hysterioides*	AB524622	AB524481	AB539115	AB539102
*Roussoella pustulans*	AB524623	AB524482	AB539116	AB539103
*Sydowia polyspora*	DQ678058	DQ678005	DQ677899	DQ677953
*Trichodelitschia bisporula*	GU348996	GU349000	GU349020	GU371802
*Trichodelitschia munkii*	DQ384096	DQ384070	–	–
*Triplosphaeria maxima*	AB524637	AB524496	–	–
*Ulospora bilgramii*	DQ678076	DQ678025	DQ677921	DQ677974
*Umbilicaria papulosa*	DQ883691	DQ883701	DQ883727	DQ883708
*Umbilicaria pustulata*	AY300839	DQ883700	DQ883726	DQ883707
*Umbilicaria spodochroa*	DQ986773	DQ986707	–	KY972682
*Usnea strigosa*	DQ973033	DQ973008	–	DQ973095
*Venturia inaequalis*	GU301878	GU296204	GU349023	–
*Vulpicida pinastri*	DQ923675	DQ912318	–	DQ912390
*Westerdykella cylindrica*	AY004343	AY016355	DQ497610	–
*Westerdykella ornata*	GU301880	GU296208	GU349021	GU371803

An ML tree involving 1000 pseudoreplicates was generated by IQ-TREE v1.6.6 (Nguyen et al. 2015) using the shared set of 3 or 4 genes. For this analysis, the best-fit substitution model was selected using ModelFinder (Kalyaanamoorthy et al. 2017), which identifies optimal model of sequence evolution (SE) by combining substitution models (e.g. GTR) with flexible rate heterogeneity across sites model. By allowing the tree topology to vary during the search for an optimal model of SE, ModelFinder reduces the chance of entrapment in local optima during model selection. GTR + F + I + G4 was selected as our best model.

The Bayesian analyses were performed in MrBayes (Ronquist et al. 2012) assuming a general time reversible model including estimation of invariant sites and a discrete gamma distribution with six rate categories (GTR + I + G) for the single-genes and the combined analyses. A run with 5.0 million generations to ensure the average standard deviation of split frequencies lower than 0.01 and employing 20 simultaneous chains was executed. Posterior probabilities above 90% and bootstrap support above 50% are considered significant supports. Every method of analysis for the single-genes and the combined analysis resulted in basically the same tree.

Phylogenetic trees were drawn using FigTree v1.4.2 (Rambaut 2012). The alignments and trees were deposited in TreeBase (http://treebase.org).

## RESULTS

### Phenotypic analyses

**Fig. 1:**
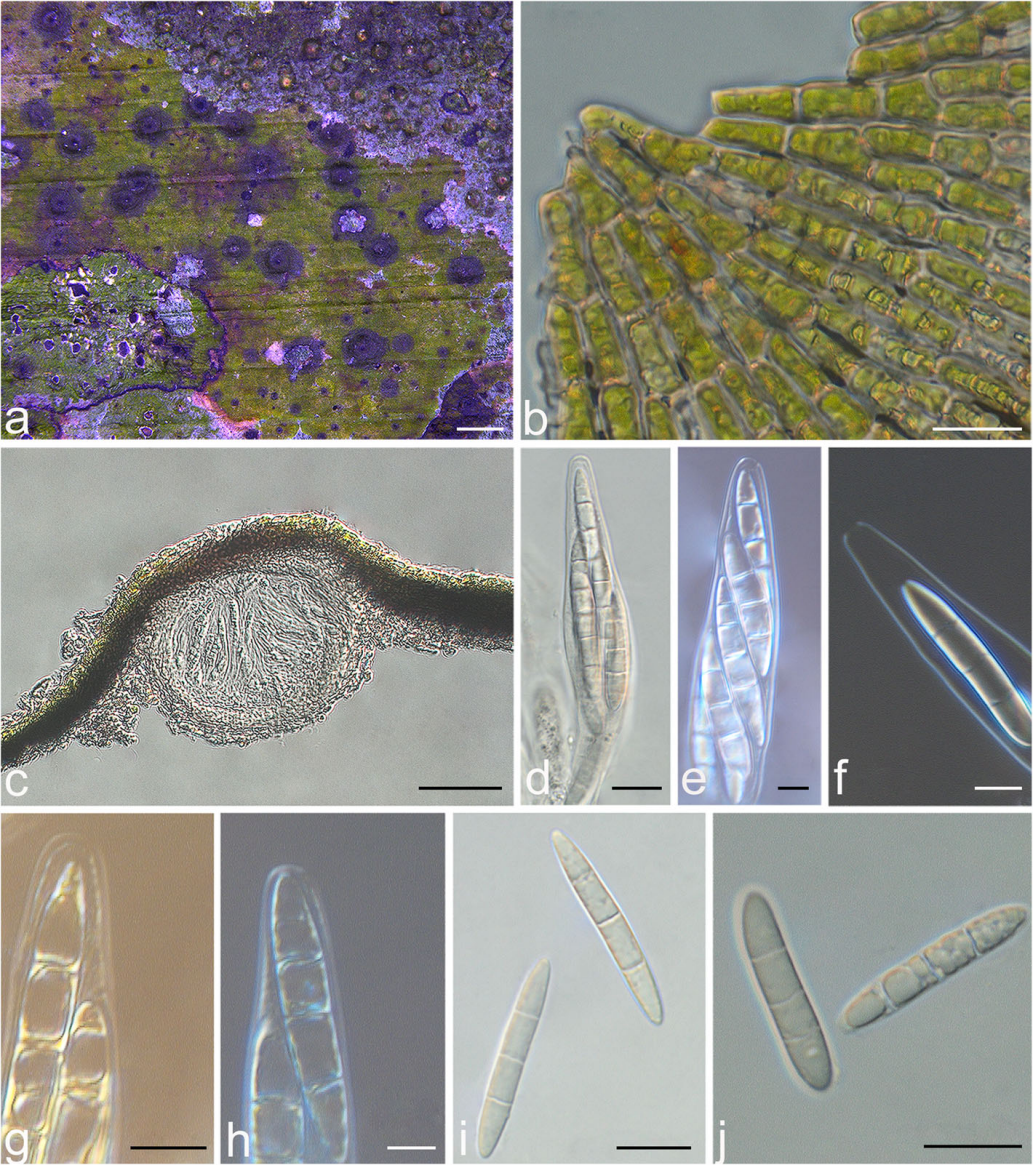
*Tenuitholiascus porinoides* (HMAS–L0139638). **a** Thallus. **b** The *Phycopeltis* algal partner. **c** Perithecia in vertical section. **d** Ascus (HMAS–L0139639). **e** Ascus (HMAS–L0139640). **f** Ascus apex (HMAS–L0141346). **g** Ascus with iodine reaction (HMAS–L0139638). **h** Ascus with iodine reaction (HMAS–L0141348). **i** Ascospores (HMAS–L0139639). **j** Ascospores (HMAS–L0139638). Scale bars: **a** = 300 μm, **b** = 10 μm, **c** = 20 μm, **d**, **i**, **j** = 10 μm, **e**–**h** = 5 μm

**Fig. 2:**
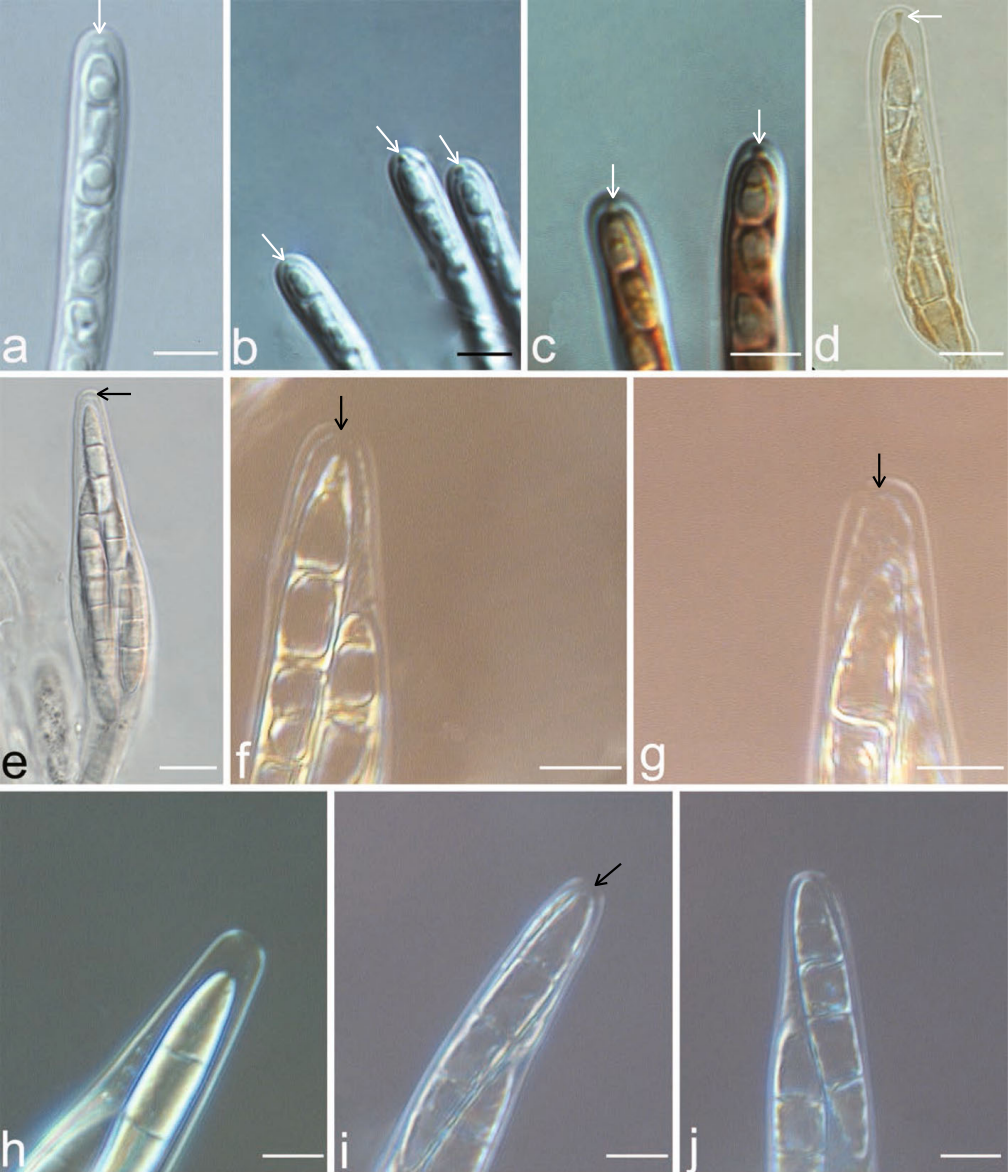
*Strigula nitidula* (HMAS–L0139358): **a**–**b** Ascus. **c** Ascus showing iodine reaction in Lugol’s solution. *Strigula* cf. *smaragdula* GD2015025–5 (HMAS–L0138067): **d** Ascus showing iodine reaction in Lugol’s solution. *Tenuitholiascus porinoides* (**e**–**g** HMAS–L0139638; **h**–**j** HMAS–L0141348) **e** Ascus. **f**–**g** Ascus with iodine reaction in Lugol’s solution. **h** Ascus. **i**–**j** Ascus with iodine reaction in Lugol’s solution. White arrows indicate the ocular chamber, and black arrows indicate the non-amyloid dome. Scale bars: a–c = 5 μm, d = 10 μm, e–j = 5 μm

In the new lineage discovered on Hainan island, the thallus was supracuticular and easily separated from the leaf surface and had a *Phycopeltis-*like photobiont (Fig. 1). The asci were bitunicate in structure, but the ascus apex differed from that of *Strigula* in lacking a thickened tholus and ocular chamber; instead, the ascus apex had an inconspicuous non-amyloid dome. The ascospores were oblong, 3-septate, with thin septa and walls, and colorless (Figs. 1, 2). This taxon was therefore considered different from any of the five genera currently recognized within *Strigulales:* from all five genera in the ascus type, from most species of *Strigula* (except the *S. phyllogena* group) in the supracuticular growth, from *Flavobathelium*, which produces similar ascospores, in the general habit, with exposed perithecia, from *Phyllocratera*, which looks superficially similar, in the very different ascospores, from *Phyllobathelium* in general habit (exposed perithecia) and ascospores, and from *Oletheriostrigula* in the lichenized habit and the ascospore type (Huhndorf and Harris 1996; Lücking 2008). These differences, together with the deviating ascus type, not only rendered the new lineage different from *Strigulaceae* but also implied an unknown position within *Ascomycota*, with some features also pointing to genera such as *Porina* in *Lecanoromycetes* (see below).

### Genotypic analyses

The dataset, including 19 LSU sequences, 16 SSU sequences, 19 TEF1-α sequences, and 19 RPB2 sequences newly generated for this study, was complemented with other sequences from different classes retrieved from GenBank (Table 3).

**Fig. 3:**
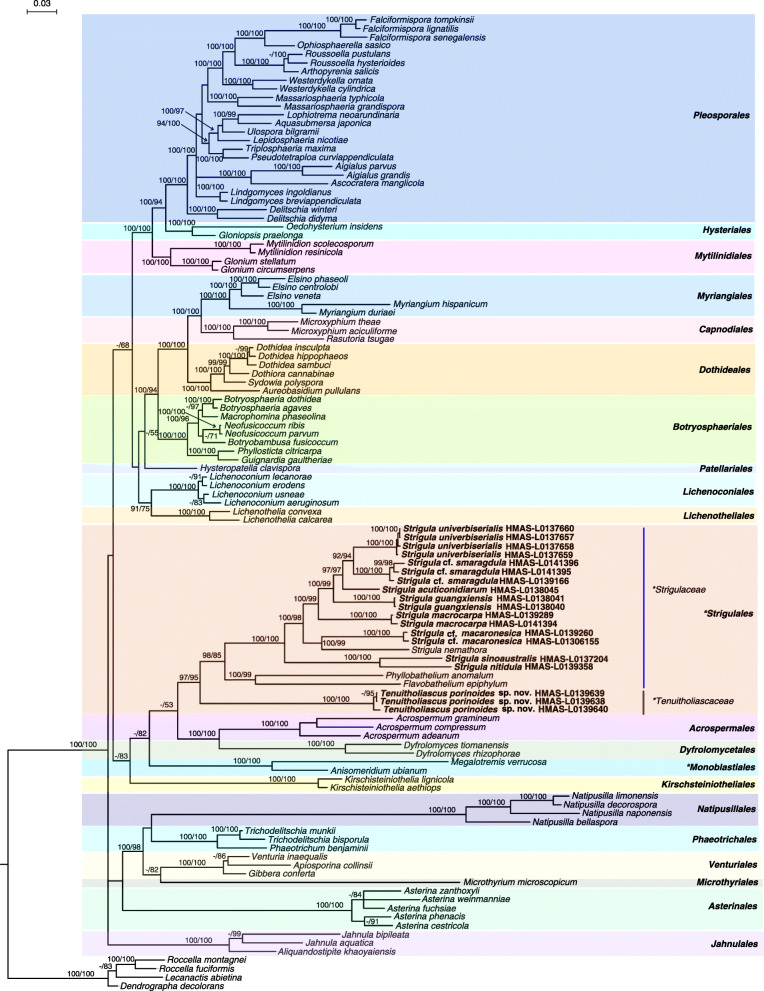
Phylogenetic tree constructed from Bayesian analyses in *Dothideomycetes* based on three gene (SSU, LSU, TEF1-α) sequences with 3001 bp. Bayesian inference posterior probabilities above 90% (left) and Maximum likelihood bootstrap probabilities above 50% (right) are shown at nodes (B–PP / ML–BP). The families and orders including lichenized taxa are marked with *. The tree was rooted to *Arthoniomycetes* spp.

For the concatenated analysis of the three selected markers, SSU, LSU, and TEF1-α, the individual datasets did not show supported conflicts, and so the three loci were combined. The resulting tree showed the new lineage in a well-supported sister group relationship with *Strigulaceae* (Fig. 3), with the following groups forming further external lineages in a supported clade: *Acrospermales*, *Dyfromycetales*, *Monoblastiales*, and *Kirschsteiniotheliales* (Fig. 3). The relationships between these lineages were not supported, except for *Acrospermales* and *Dyfromycetales* forming a strongly supported clade. This appears to be the first study that places the lichenized *Monoblastiales* rather close to *Strigulales*, which is notable as both clades share important characters and have been considered closely related or even belonging in the same family in the past (e.g. Harris 1975; Lücking 2008).

To assess placement of the new lineage within *Dothideomycetes*, a dataset consisting of four loci (SSU, LSU, TEF1-α, and RPB2) was also constructed and analysed (Additional file 1). It is evident that the new lineage is a member of the class *Dothideomycetes*, rather than *Lecanoromycetes*. Also in this analysis, the specimens of the new genus formed a separate clade supported sister to the known genera of *Strigulales*.

**Fig. 4:**
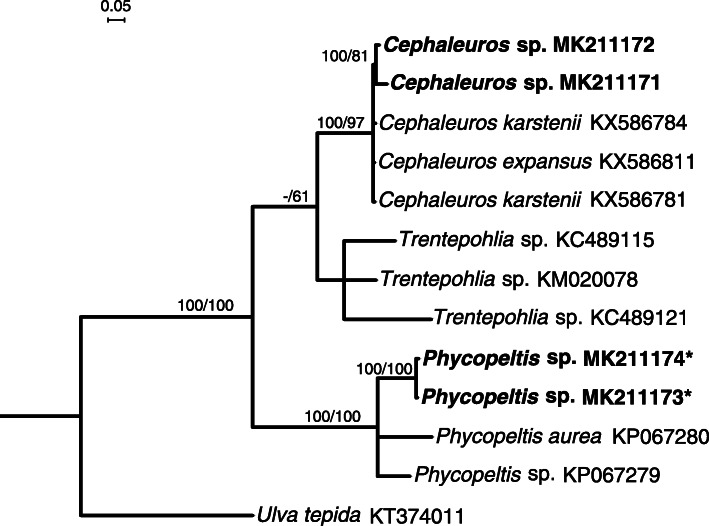
Phylogenetic tree constructed from Bayesian analyses based on ITS of photobionts of *Trentepohliaceae*. Bayesian posterior probabilities (B–PP) > 90%, and Maximum likelihood bootstrap proportions >50% are shown at nodes (B–PP / ML–BP). The new sequences generated in this study are in bold, and algal partners from *Tenuitholiascus porinoides* are marked with *

In the ITS tree of the analysed photobionts, the photobiont of the new lineage clustered with algae identified as *Phycopeltis*, with *Cephaleuros* and *Trentepohlia* forming separate branches with high support (Fig. 4), supporting our phenotypic assessment of the photobiont as *Phycopeltis*.

## DISCUSSION

The phenotype and molecular data demonstrate that the material from Hainan island represents a previously unknown lineage of foliicolous lichens which merits the status of a new genus and family. The lineage is closely related to *Strigulaceae* but differs clearly in the ascus type. Phenotypically, the new taxon bears some resemblance with members of *Strigulaceae*, but also with some unrelated lichenized lineages in the *Lecanoromycetes* and *Eurotiomycetes*. Thus, the general habit with supracuticular growth, a *Phycopeltis* photobiont, and exposed perithecia resembles that of the *Strigula phyllogena* group (Lücking 2008) and of *Phyllocratera* (Lücking and Sérusiaux 2013), whereas the ascospores are similar to those of *Flavobathelium* (Lücking 2008). The comparatively thin-walled asci, together with the unbranched paraphyses and the oblong, thin-walled, 3-septate ascospores would place the new taxon close to *Porina*, in particularly the black-fruited species also recognized in the genera *Pseudosagedia* and/or *Trichothelium* (Harris 1995; Lücking 2008; Lücking et al. 2017; Sobreira et al. 2018). Among *Porina* s.lat., the most similar foliicolous species is *P. chrysophora* (Santesson 1952), which agrees in the black, hemispherical perithecia and the 3-septate ascospores, but differs in the dispersed thallus, the absence of a basally expanding perithecial wall, and the much smaller ascospores. Some foliicolous species of *Phylloblastia* (*Eurotiomycetes*: *Verrucariales*) previously classified in the genus *Pocsia* (Lücking 2008) are also superficially similar and may produce 3-septate ascospores; however, they are easily set apart by the lack of paraphyses, the apically thick-walled asci, and the different photobiont usually consisting of more rounded cells in irregular arrangement.

The apical ascus structure in all previously recognized members of *Strigulales* is the so-called *Strigula*-type, characterized by structurally bitunicate asci with a short tholus and small ocular chamber (Fig. 2a–d). The asci of the new lineage are similar to those of *Strigulales* in being bitunicate, but differ in their apical structure, in that the asci have an inconspicuous, non-amyloid dome lacking an ocular chamber (Fig. 2e–j). In some stages of development, the inner wall layer becomes gradually thinner and makes the asci appear unitunicate, similar to the genus *Porina* (Fig. 2h, j).

Although the overall features of the new genus show affinities to other genera currently included in *Strigulaceae,* the difference in ascus structure is more fundamental and merits recognition of the new taxon at a rank higher than genus. We even pondered the possibility establishing a separate order, but felt this level too high considering that likely many other lineages in this assemblage await discovery. Even foliicolous lichens remain much understudied, as illustrated by the fact that the new genus was quite abundant in the type locality and was collected multiple times during a single day. Also, there are other examples in which taxa with deviating ascus types are classified within a single order, such as *Baeomycetales* and *Lecanorales* within *Lecanoromycetes* (Lumbsch et al. 2007; Miadlikowska et al. 2014).

Our three-locus based analyses (Fig. 3) provided an important insight into the phylogenetic adscription of the order *Strigulales* amongst *Dothideomycetes,* the members of which are generally characterized by thin interascal filaments. Our phylogeny largely matches that of Hyde et al. (2013) in the close relationship of *Strigulaceae* with *Acrospermaceae* (*Acrospermales*), *Dyfrolomycetaceae* (*Dyfrolomycetales*), and *Kirschsteiniotheliaceae* (*Kirschsteiniotheliales*). All four families clustered with strong support in a single clade, which also included the new lineage close to *Strigulaceae* and the *Monoblastiaceae* (*Monoblastiales*). The latter comes as surprise, as this family had not been recovered as not closely related in other analyses (Nelsen et al. 2009, 2011a; Hyde et al. 2013). All families have different morphologies and life styles, *Tenuitholiascaceae* being closest to *Strigulaceae* in these aspects but differing from all other lineages in the ascus type. Ascospores in the new lineage are most similar to those of *Strigulaceae* (*Flavobathelium*); *Arcospermaceae* have filiform spores, *Kirschsteiniotheliaceae* 1-septare but brown spores, and *Dyfrolomycetaceae* muriform spores (somewhat similar to *Phyllobathelium* but in shape more similar to those of *Strigula*). We therefore propose to recognize the new genus under a new family within *Strigulales*.

## TAXONOMY

***Tenuitholiascaceae*** S.H. Jiang, Lücking & J.C. Wei, ***fam. nov.*** — *Fungal Names* FN570578;

*Type*: *Tenuitholiascus* S.H. Jiang, Lücking & J.C. Wei.

*Diagnosis*: Distinguished from *Strigulacaeae* in the structure of the ascus apex, which lacks the short tholus and ocular chamber characteristic of *Strigula*-type asci. In the nuLSU alignment (Additional file 2), the following positions are consistently diagnostic at family level: 113 (A vs. G), 143 (A vs. G), 166 (C vs. G), 168 (G vs. T), 207–208 (AT vs. GC), 313 (T vs. C), 363–364 (TC vs. GT), 377 (A vs. G), 385 (T vs. C), 397 (A vs. C), 449 (T vs. G), 484–485 (TG vs. CC), 505 (T vs. C).

***Tenuitholiascus*** S.H. Jiang, Lücking & J.C. Wei, ***gen. nov.*** — *Fungal Names* FN570581;

*Etymology*. From the Latin tenuis- (slender), the Latin tholus (dome), and the Latin ascus (tube, bag), conveys an important feature of the apically thin-walled asci.

*Type*: *Tenuitholiascus porinoides* S.H. Jiang, Lücking & J.C. Wei.

*Diagnosis*: The only genus of the family, distinguished from *Strigulaceae* in ascus structure (see above), from the *Strigula phyllogena* group also in the 3-septate, oblong ascospores, from *Phyllocratera* in the small, 3-septate ascospores, and from *Flavobathelium* in the external habit with exposed perithecia.

***Tenuitholiascus porinoides*** S.H. Jiang, Lücking & J.C. Wei, ***sp. nov.*** — *Fungal Names* FN570580; Fig. 1

*Etymology*. The specific epithet conveys the similarity with the genus *Porina*, although not related to the latter.

*Type*: **China:** *Hainan*: Changjiang county, Bawangling National Nature Reserve, 19°07′07″N, 109°09′12″E, alt. 700 m, on living leaves, 4 Sept. 2017, *S.H. Jiang HN20171851* (HMAS–L0139638 – holotype).

*Description*: Thallus supracuticular, easily separated from the leaf surface, continuous, smooth, pale green, 3–12 mm diam, 30–52.5 μm thick. *Algal partner*: *Phycopeltis*, cells rectangular, 8–14 × 3–5 μm, composed of anastomosing filaments lying in one layer and forming regular radial plates or irregular nets. *Ascomata* perithecia, globose, scattered or clustered, exposed but covered by thin thallus layer up to the ostiole, central part wart-shaped, sometimes basal part broadly spreading to form horizontal plate, 0.25–0.5 mm diam and 80–150 μm high, greyish black. *Involucrellum* carbonized, black, 55–125 μm thick. *Exciple* dense, prosoplectenchymatous, 10–12.5 μm thick, colourless to brown. *Interascal filaments*: unbranched or simply branched, thin. *Asci* bitunicate in structure, apex with a non-amyloid rounded, sometimes appearing almost unitunicate in some developmental stages, due to the gradually thinner inner walls (Fig. 1f; Fig. 2h, j), clavate to cylindrical, 75–90 × 10–12.5 μm, I–, KI–, 8-spored. *Ascospores* fusiform, 3-septate, colourless, 25–30 × 6–8 μm. *Pycnidia* common, wart-shaped, immersed to erumpent, 0.05–0.1 mm diam, black. *Conidia* (microconidia) fusiform, hyaline, non-septate, 4–5 × 1.5–2 μm.

*Alga partner*. The trentepohlioid genera *Cephaleuros*, *Phycopeltis* and *Trentepohlia* have been reported from *Strigulaceae* (Lücking 2008; Nelsen et al. 2011b). In addition to morphology, four newly generated ITS sequences of the photobiont were aligned with selected *Trentepohliaceae* from GenBank; the selected sequences of *Cephaleuros*, *Phycopeltis* and *Trentepohlia* formed separate branches each (Fig. 4), and the photobiont of the new lineage clustered with *Phycopeltis*.

*Chemistry*: No substances detected by TLC.

*Ecology and distribution*: At present*,* the new species is known only from the type locality (Hainan island) in China, where it grows on leaves in wet tropical forest.

*Remarks*: For similarities and differences of the new species with other taxa in *Strigulaceae* and the unrelated genera *Porina* and *Pocsia*, see above.

*Other specimens examined*: **CHINA:** *Hainan*: Changjiang county, Bawangling National Nature Reserve, 19°07′07″N, 109°09′12″E, alt. 700 m, on living leaves, 4 September 2017, *S.H. Jiang HN20171719* (HMAS–L0141342), *HN20171740* (HMAS–L0141344), *HN20171808* (HMAS–L0141348), *HN20171820* (HMAS–L0141343), *HN20171826* (HMAS–L0141349), *HN20171844* (HMAS–L0141346), *HN20171845* (HMAS–L0139639), *HN20171850* (HMAS–L0141345), *HN20171857* (HMAS–L0139640), *HN20171875* (HMAS–L0141347).

## CONCLUSIONS

Molecular data of some foliicolous lichens collected in Hainan island revealed a new lineage morphologically similar to *Porina* but phylogenetically related to *Strigulaceae*, differing from the latter in ascus type, which merits the status of a new genus (*Tenuitholiascus*) and family (*Tenuitholiascaceae*) within *Strigulales*. The extent of lack of exploration on tropical foliicolous lichens in Asia is indicated by the new genus having been collected 11 times on a single day. Indeed, the discovery of this previously unsuspected lineage is an example of how little we know. It should still be stressed that the effort to complete the inventory of tropical lichens should be made in the future.

## Supplementary information

**Additional file 1. **Phylogenetic tree constructed from Bayesian analyses in *Dothideomycetes* and *Lecanoromycetes* based on four gene (SSU, LSU, TEF1-α, and RPB2) sequences with 4033 bp. Bayesian inference posterior probabilities above 90% (left) and Maximum likelihood bootstrap probabilities above 50% (right) are shown at nodes (B–PP / ML–BP). The families and orders including lichenized taxa are marked with *. The tree was rooted to *Basidiomycota.*

**Additional file 2. **Alignment of the nuLSU for *Strigulaceae* and *Tenuitholiascaceae* to discern diagnostic positions at family level.

## Data Availability

The materials are available as Additional files 1 and 2. All sequence data generated for this study (Tables 1 and 2) can be accessed via GenBank: https://www.ncbi.nlm.nih.gov/genbank/. Alignments are available at TreeBase (http://www.treebase.org).

## Abbreviations

B–PP: Bayesian posterior probabilities
ML: Maximum likelihood
ML–BP: Maximum likelihood bootstrap probabilities
RPB2: The second largest subunit of RNA polymerase II
TEF1-α: Translation elongation factor 1 α
