# Supplementary figures and images for: A new genus and species of foliicolous lichen in a new family of Strigulales (Ascomycota: Dothideomycetes) reveals remarkable class-level homoplasy
Source: IMA Fungus. 2020 Feb 5;11:1. doi: 10.1186/s43008-019-0026-2 (PMC7325298; doi:10.1186/s43008-019-0026-2)

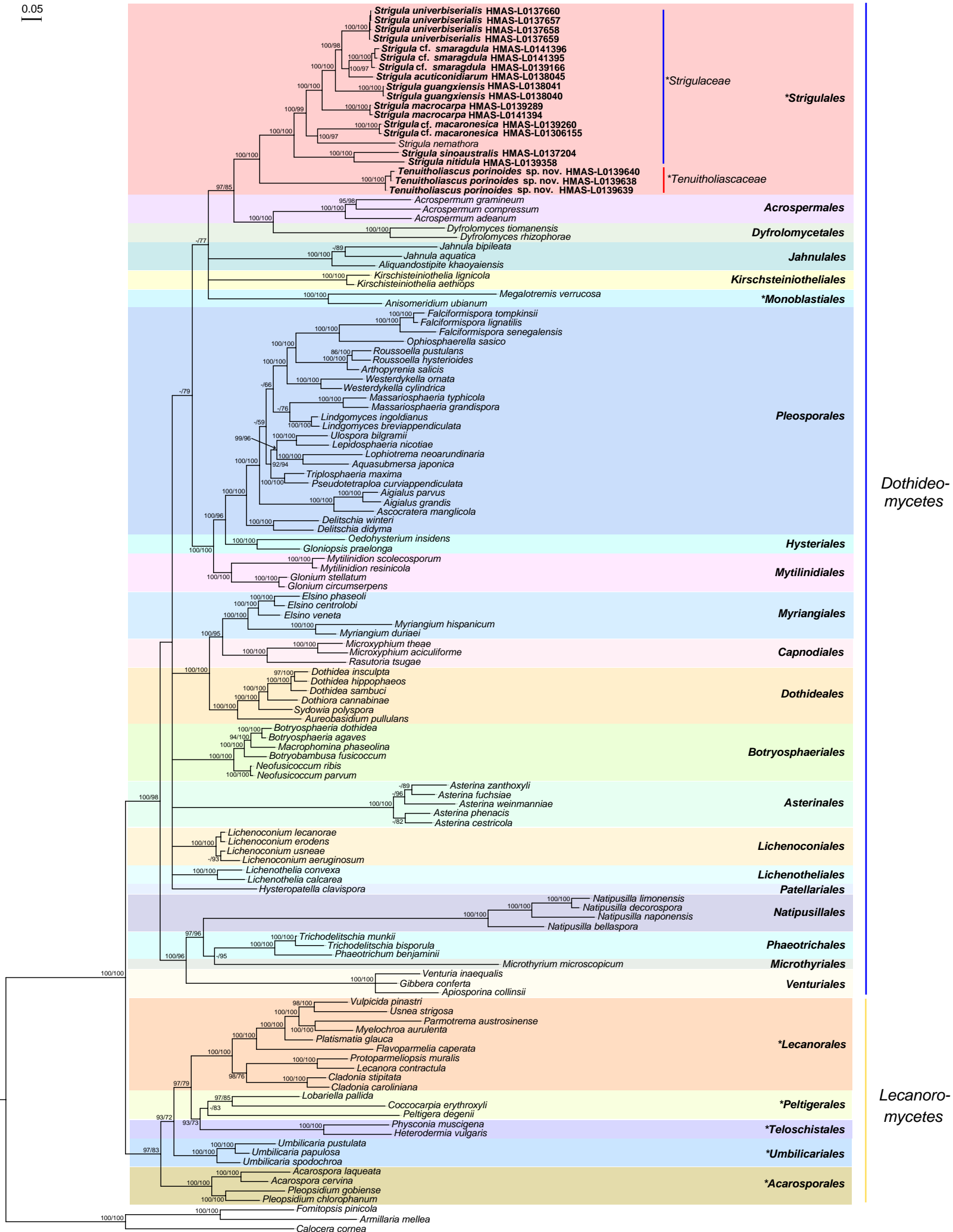Dothideo-  
mycetesLecanoro-  
mycetes

Supplement: Supplementary file 1 — Additional file 1. Phylogenetic tree constructed from Bayesian analyses in Dothideomycetes and Lecanoromycetes based on four gene (SSU, LSU, TEF1-α, and RPB2) sequences with 4033 bp. Bayesian inference posterior probabilities above 90% (left) and Maximum likelihood bootstrap probabilities above 50% (right) are shown at nodes (B–PP / ML–BP). The families and orders including lichenized taxa are marked with *. The tree was rooted to Basidiomycota. [file 43008_2019_26_MOESM1_ESM.pdf]
